# Is crystalloid cardioplegia a strong predictor of intra-operative hemodilution?

**DOI:** 10.1186/1749-8090-9-23

**Published:** 2014-01-27

**Authors:** Murat Günday, Hakan Bingöl

**Affiliations:** 1 Department of Cardiovascular Surgery, Baskent University Faculty of Medicine, Ankara, Turkey; 2 Department of Cardiovascular Surgery, Ankara Çankaya Hospital, Aşağı Dikmen mah. 575 sok, Orankent konutları B blok No:12, OR-AN Çankaya, Ankara, Turkey

**Keywords:** Cardioplegia, Crystalloid, Coronary artery, Bypass, Hemodilution

## Abstract

**Introduction:**

Complications due to hemodilution (hematocrit value less than 22%) after cardiopulmonary bypass inevitably resulted with significantly greater intensive care requirements, long hospital stays, more operative costs, and increased mortality rates. We tried to identify whether crystalloid cardioplegia is the strongest predictor of intraoperative hemodilution or not.

**Materials and methods:**

One hundred patients were included into this randomized prospective study. Patients were divided into the two groups. Crystalloid cardioplegia were given to the odd-numbered patients (Group 1, n = 50 patients) and blood cardioplegia were given to the even-numbered patients (Group 2, n = 50 patients). St. Thomas-II solution was used in Group-1 and Calafiore cold blood cardioplegia was in Group-2.

**Results:**

Average intraoperative hematocrit value was 18.4% ± 2.3 in crystalloid group 24.2% ±3.4 in blood cardioplegia group (p < 0.001). The lowest hematocrit value was 15% and 20% in two groups respectively (p < 0.001). In crystalloid group average intraoperative packed red blood cell (RBC) transfusion was 2.3 ± 0.41 units, 0.7 ± 0.6 units blood cardioplegia group (p = 0.001). Average transfused RBC was 2.7 ± 0.8 units in crystalloid group, 0.9 ± 0.4 units blood cardioplegia group (p < 0.001). Multivariate analyses confirmed age (p = 0.005, OR = 3.78), female gender (p = 0.003, OR = 2.91), longer cross-clamp time (>60 minutes) (p = 0.001, OD = 0.97), body surface area <1.6 m^2^ (p = 0.001, OR = 6.01) and crystalloid cardioplegia (p < 0.001, OR = 0.19) as predictor of intraoperative hemodilution.

**Conclusion:**

Crystalloid cardioplegia, compared to blood cardioplegia not only causes much more intra-operative hemodilution but also increases the blood transfusion requirement. Hemodilution and increased transfusion increases the intensive care unit and hospital stay, in the early postoperative period.

## Background

The effect of hemodilution during cardiopulmonary bypass is extensively studied and lower hematocrit levels (< 22%) caused postoperative organ failure [1]. Renal failure, stroke, perioperative myocardial infarction, prolonged mechanical ventilation and multiorgan failure are much more common in hemodilution conditions [2]. There are several predictors of hemodilution. Prebypass hematocrit values (such as anemia), female gender, Cardiopulmonary bypass (CPB) time, age, renal failure, and small body surface area (BSA) are the strong predictor of hemodilution and consequently blood transfusion [3-5]. We know that allogenic blood transfusion increases the risk of mortality [6] and morbidity such as pulmonary edema [7], renal failure [8], transfusion complications [9]. There are lots of surgeons still using Crystalloid Cardioplegia for its simplicity, economy and fairly good efficacy in the vast majority of patients. Nevertheless, Crystalloid Cardioplegia presents major disadvantages compared with the same volume of blood cardioplegia [10]. It poorly supplies oxygen; it increases the degree of hemodilution which is already high after circulation of the priming volume and it decreases the oncotic pressure [11]. As we mentioned negative effects of hemodilution and blood transfusion are clarified. We have prospectively investigated the effects of Crystalloid Cardioplegia on hemodilution and necessity of blood transfusion by comparing with blood cardioplegia.

## Methods

Between 2010–2013, One hundred patients were included into our randomized prospective study. We decided to give Crystalloid cardioplegia to the odd-numbered patients and blood cardioplegia to the even numbered patients. Informed consents were obtained from all patients. Institutional board approval from ethical committee of hospital (Süleyman Demirel University, Faculty of Medicine) was also obtained (November 13, 2006/3). Crystalloid cardioplegia (St. Thomas-II solution) have been used in Group-1. Fifteen ml/kg St-Thomas II solution was given both antegrade and retrograde initially. Then in every 20 minutes St-Thomas II solution was given retrograde (7 ml/kg). Just before cross-clamp removal 150 ml St-Thomas II and 250 ml blood were given antegradely (Hot shot). Calafiore blood cardioplegia was used in group-2 patients [12,13]. This solution was given at the 600 ml warm solution and 600 ml cold solution in the induction period then 600 ml cold solution in every 20 minutes both antegradely and retrogradely. Just before cross clamp removal 600 ml warm blood cardiplegia (Hot Shot) with lower potassium (4 mmol) was given antegradely (Table 1). Patients’ characteristics were summarized in Table 2.

**Table 1:** Ingredients of calafiore cold blood cardioplegia solution

		
INDUCTION	Native blood; 1000 ML Ringer solution; 70 mmol K^+^; 30 ml 8.4% NaHCO_3_; ratio “4/1	600 ml warm solution; 600 ml cold solution
MAINTENANCE	Same additives with induction	600 ml cold solution
HOT SHOT	4 mmol K+	600 ml warm solution

**Table 2:** Preoperative characteristics of patients

**Variable**	**Crystalloid cardioplegia [no = 50]**	**Blood cardioplegia [no = 50]**	**p value**
Age*	61 ± 12	63 ± 9	0.651
Male	37	39	0.466
Smoking	34	29	0.012
COPD	12	14	0.834
Hypertension	25	21	0.543
Congestive heart failure (no)	2	3	0.335
Peripheral vascular disease	11	9	0.746
Cerebral vascular disease	6	7	1.00
Average body surface area (BSA m^2^)	1.91 ± 0.5	1.89 ± 2.2	0.855
Body mass index (kg/m^2^)*	33.4 ± 4.2	29.3 ± 6.8	0.455
Ejection fraction (%)*	45.6 ± 7.8	50.1 ± 3.6	0.023
Preoperative IABP	2	-	0.242
Number of diseased vessels* (range 1–4)	2.1 ± 0.4	1.9 ± 0.6	0.744
LMCA disease (>50%)	5	3	0.721
Previous MI	31	37	0.462
Previous PTCA or stent	8	11	0.631
Preoperative hematocrit value (%)	38.5 ± 3.7	40.4 ± 5.6	0.563
Preoperative hemoglobine value (gr/dL)	13.3 ± 2.3	13.9 ± 2.9	0.646

*mean ± SD.

*Abbrevations*: *COPD* chronic obstructive pulmonary disease, *IABP IABP* intra-aortic balloon pump, *LMCA* left main coronary artery, *MI* myocardial infarction, *PTCA* Percutaneous transluminal balloon angioplasty.

### Operative characteristics

All patients in both groups had elective operations. All patients underwent primary coronary revascularization. CPB was done in all patients with two stage venous and aortic cannula. Moderate hypothermia (26˚C) and topical cooling with iced saline slush were used in all patients. Myocardial preservation was accomplished as mentioned previously in both groups. CPB was established with a roller pump, non-pulsatile flow after anticoagulation with bovine lung heparin (3 mg/kg) and activated clotting time was maintained for more than 480 s. Membrane oxygenators (Capiox-E Terumo Corp, Tokyo, Japan) were used. Heparin was neutralized by protamine (3.5 mg/kg) at the end of the CPB. All distal anastomoses were done in a single cross-clamp period.

### Transfusion protocols

Packed red blood cell transfusion was used when hematocrit (Htc) levels were less than 22% during the intra-operative period. Mostly Packed red blood cell (RBC) added pump reservoir during the CPB period. Transfusion was done when hematocrit < 18% in the postoperative period. In older patients (>70 years old) Hct levels <20% was accept as the lowest limit. Platelet transfusion and fresh frozen plasma was limitedly used in both groups. Fluid regime strictly followed during the postoperative periods.

### Sample size

According to our previous clinical observations, crystalloid cardioplegia reduces intraoperative Htc levels below 25% in coronary artery bypass graft (CABG) patients. We predicted 10% reduction of Htc levels in blood cardioplegia patients. To detect this difference with 95% confidence limits and power of 80% we needed to enter at least 49 patients into each group [14].

### Exclusion criteria

All re-operative and urgent and emergency procedures, reoperations, bleeding disorders, dialysis dependent renal failure, and anemic patients (preoperative Hct < 20%) were excluded.

### Statistical analyses

Statistical analysis was performed with SPSS software version 10.0 (SPSS Inc., Chicago, IL). Clinical data were expressed as mean values ± standard deviation. Differences were analyzed with Fisher’s exact test, *X*^2^-test, unpaired Student’s *t*-test and Mann–Whitney test. We investigated the effects of the variables on hemodilution and transfusion requirement by calculating odds ratios (OR) in univariate analyses for all CABG patients. Variables for which the unadjusted *p*-value was ≤0.20 in logistic regression analysis were identified as potential risk markers and included in the full model. We conducted stepwise multivariate analyses by using logistic regression. We reduced the model by using backward elimination and we eliminated potential risk markers by using likelihood ratio tests.

## Results

Average CPB time was 87 ± 14 minutes in crystalloid group, 94 ± 11 in blood cardioplegia group (p = 0.301). Average cross clamp time was 61 ± 15 and 58 ± 23 minutes in both groups respectively (p = 0.433). Low cardiac output developed in 11 patients in crystalloid and 5 patients in blood cardioplegia group (p = 0.044). Positive inotropic drugs were used in 17 patients in crystalloid cardioplegia group, 8 patients in blood cardioplegia group (p = 0.032). Intra-aortic balloon was inserted to five patients in crystalloid group and only one patient in blood cardioplegia group. Dialysis required renal failure was developed only one patient in crystalloid cardioplegia group. Four patients required a second look for hemorrhage in crystalloid group and two patients in blood cardioplegia group (p = 0.677). Radial artery was used in 17 patients in group 1 and 19 patients in group 2. LITA was used 45 patients in group 1 and 47 patients in group 2. Average intraoperative hematocrit (%) value was 18.4 ± 2.3 in crystalloid group 24.2 ± 3.4 in blood cardioplegia group (p < 0.001). The lowest Htc value was 15% and 20% in both groups respectively (p < 0.001). 2.3 ± 0.41 units packed RBC transfusion was necessary intra-operatively in crystalloid group, 0.7 ± 0.6 units in blood cardioplegia group (p = 0.001). Average transfused RBC was 2.7 ± 0.8 units in crystalloid group, 0.9 ± 0.4 units blood cardioplegia group (p < 0.001). Mean intensive care unit (ICU) stay was 2.7 ± 1.2 days in crystalloid group, 1.1 ± 0.45 days in blood cardioplegia group (p = 0.001). Mean hospital stay was 8.6 ± 2.1 versus 6.4 ± 1.1 days in respectively (p < 0.001). Two patients died in crystalloid group but none in blood cardioplegia group (p = 0.494). Perioperative and postoperative variables were summarized in Table 3.

**Table 3:** Perioperative and postoperative variables

**Variable**	**Crystalloid cardioplegia [no = 50]**	**Blood cardioplegia [no = 50]**	**p value**
CPB time (min)	87 ± 14	94 ± 11	0.301
Cross-clamp time	61 ± 15	58 ± 23	0.433
Distal anastomosis (range 1–4)	3.4 ± 0.9	3.1 ± 0.4	0.244
Low cardiac output	11	5	0.044
IABP	5	1	0.031
Positive inotropic drug requirement	17	8	0.032
Renal failure need dialysis	1	-	1
Revision			
Tamponade	1	1	-
Bleeding	3	1	0.677
Ventilation >48	2	1	1.00
Sternal dehissence	3	-	0.242
TIA	1	1	-
Stroke	1	-	-
Sternal wound infection	2	1	-
Mediastinitis	1	-	-
Internal thoracic artery			
-None	2	3	1.00
-Single	45	47	0.751
-Double	3	-	
Radial artery	17	19	0.835
Saphenous vein	50	50	1.00
Average intraoperative Htc (%)	18.4 ± 2.3	24.2 ± 3.4	<0.001
Nadir intraoperative Htc (%)	15	20	<0.001
Average intraoperative Hb (gr/dL)	5.5 ± 1.2	6.7 ± 1.3	0.001
Average intraoperative transfusion of Packed RBC	2.3 ± 0.41	0.7 ± 0.6	0.001
Average transfusion of packed RBC	2.7 ± 0.8	0.9 ± 0.4	<0.001
Average FFP transfusion	2.1 ± 0.2	1.2 ± 0.8	0.001
Average platelet transfusion	1.2 ± 0.3	-	
Mortality	2	-	0.494
ICU stay (days)	2.7 ± 1.2	1.1 ± 0.45	0.001
Hospital stay (days)	8.6 ± 2.1	6.4 ± 1.1	<0.001
SVT	29	21	0.008

*Abbrevations*: *CPB* cardiopulmonary bypass, *IABP* intra-aortic balloon pump, *TIA* transient ischemic attack, *Htc* Hematochrit, *Hb* Hemoglobine, *RBC* Red blood cell, *FFP* fresh frozen packed, *ICU* intensive care unit, *SVT* supraventricular tachycardia.

### Predictors of intra-operative hemodilution

Data for all groups were combined and 11 variables were subjected to statistical analysis as a predictor of intraoperative hemodilution (Htc < 22%). As shown in Table 4, univariate analysis identified age (p = 0.009, OR = 2.07), female gender (p = 0.007, OR = 0.89), longer cross-clamp time (>60 minutes) (p = 0.046, OD = 0.97), BSA <1.6 m^2^ (p = 0.005, OR = 5.60) and crystalloid cardioplegia (p < 0.001, OR = 0.36) as predictors of intraoperative hemodilution. The value of -2 log likelihood of multivariate analyses was 421.504. Multivariate analyses confirmed five of six variables. These were age (p = 0.005, OR = 3.78), female gender (p = 0.003, OR = 2.91), longer cross-clamp time (>60 minutes) (p = 0.001, OD =0.97), BSA <1.6 m^2^ (p = 0.001, OR = 6.01) and crystalloid cardioplegia (p < 0.001, OR = 0.19).

**Table 4:** Effects of various variables on the intraoperative hemodilution (Htc < 22) based on univariate and multivariate logistic regression analyses

	**Univariate**			**Multivariate**		
	**Unadjusted odds ratio**	**95% CI**	**p value**	Adjusted* odds ratio	**95% CI**	**p value**
Age	2.07	1.66-3.34	0.009	3.78	2.67-4.33	0.005
Female	0.89	0.42-1.85	0.007	2.91	1.99-3.01	0.003
Diabetes	0.99	0.55-1.88	0.712	1.66	0.2-5.43	0.476
EF	0.92	0.89-0.96	0.101	1.7	0.1-7.8	0.765
CPB time	1.03	0.99-1.10	0.211	2.6	2.1-2.91	0.001
X-clamp time > 60	0.97	0.91-1.05	0.046	0.97	0.94-1.03	0.001
COPD	3.41	2.13-4.29	0.111	3.94	2.35-6.99	0.178
LITA	0.49	0.25-0.81	0.023	0.65	0.32-1.25	0.230
Radial artery	0.75	0.36-1.42	0.442	2.11	1.01-3.36	0.536
BSA < 1.60 m^2^	5.60	4.71-8.95	0.005	6.01	5.67-6.33	0.001
Crystalloid cardioplegia	0.36	0.19-0.62	<0.001	0.19	0.08-0.35	<0.001

*Adjusted for ejection fraction, cross-clamp time, LITA, COPD.

*EF* ejection fraction, *CPB* cross-clamp time, *LITA* left internal thoracic artery, *COPD* chronic obstructive pulmonary disease, *BSA* body surface area.

### Predictors of transfusion requirement of packed red blood cells

Data for all groups were combined and 11 variables were subjected to statistical analysis as a predictor of transfusion requirement of packed red blood cell. As shown in Table 5 univariate analysis identified age (p = 0.034, OR = 3.1), female gender (p = 0.004, OR = 1.12), CPB time (p = 0.006, OR = 2.33), longer cross-clamp time (>60 minutes) (p = 0.003, OD = 2.16), BSA <1.6 m^2^ (p = 0.001, OR = 3.71) and crystalloid cardioplegia (p < 0.001, OR = 1.25) as predictors of transfusion requirement. Multivariate analyses confirmed five of six variables. These were female gender (p = 0.023, OR = 3.91), CPB time (p = 0.001, OR = 1.7), longer cross-clamp time (>60 minutes) (p = 0.001, OD = 4.45), BSA <1.6 m2 (p < 0.001, OR = 3.83) and crystalloid cardioplegia (p < 0.001, OR = 0.78).

**Table 5:** Effects of various variables on the transfusion requirement of packed red blood cell based on univariate and multivariate logistic regression analyses

	**Univariate**			**Multivariate**		
	**Unadjusted odds ratio**	**95% CI**	**p value**	Adjusted* odds ratio	**95% CI**	**p value**
Age	3.1	1.11-4.34	0.034	2.3	1.67-3.53	0.065
Female	1.12	0.92-1.85	0.004	3.91	2.99-3.81	0.023
Diabetes	1.23	0.95-2.88	0.844			
EF	0.86	0.59-1.96	0.101			
CPB time	2.33	1.99-3.10	0.006	1.7	1.1-2.91	0.001
X-clamp time > 60 min	2.16	1.91-2.55	0.003	4.45	3.94-4.98	0.001
COPD	4.61	1.13-7.29	0.673			
LITA	2.34	0.25-4.81	0.123			
Radial artery	2.45	1.31-4.02	0.678			
BSA < 1.60 m^2^	3.71	3.34-4.95	0.001	3.83	3.67-5.13	<0.001
Crystalloid cardioplegia	1.25	0.91-1.62	<0.001	0.78	0.18-1.2	<0.001

*Adjusted for ejection fraction, cross-clamp time, LITA, COPD.

*EF* ejection fraction, *CPB* cross-clamp time, *LITA* left internal thoracic artery, *COPD* chronic obstructive pulmonary disease, *BSA* body surface area.

## Discussion

Cardioplegia protects the heart from ischemic injury and postoperative heart failure during cardiopulmonary arrest period. Initially crystalloid cardioplegia was introduced as an agent to allow for hypothermic hyperkalemic arrest. Various additives were explored to optimize the myocardium during the time of ischemia [15]. Although cardioplegia is generally accepted to be a mandatory tool for myocardial protection during on-pump cardiac surgery, there is still controversy regarding various aspects including its composition, temperature, and mode of delivery. Blood was then found to be the most logical vehicle for delivery of potassium cardioplegia [16,17] The optimal cardioplegic temperature, timing, and routes of delivery were further explored [18]. There are still controversies between crystalloid and blood cardioplegia for better myocardial protection. Øvrum E. and colleagues [19] did not found statistically significant differences between crystalloid and blood cardioplegia. Rinne T [20] demonstrated a higher incidence of spontaneous resumption of sinus rhythm after blood cardioplegia compared with that after crystalloid cardioplegia. But Øvrum E and colleagues [19] found no statistical differences concerning conduction disturbances. Blood as opposed to crystalloid cardioplegia could potentially improve postoperative cardiac outcomes, because it more closely approximates normal physiology caused in part by its oxygen carrying capacity and less associated hemodilution [15].

Intraoperative hemodilution and transfusion requirement becomes most critical subjects in the last decade [1-9]. Habib RH [1] specifically showed that major complications, including stroke, myocardial infarction, low cardiac output syndrome, renal failure, pulmonary edema, reoperation due to bleeding, sepsis, and multiorgan failure, were increased as the nadir hematocrit during CPB decreased. Moreover, this hematocrit–complications association inevitably resulted in significantly and systematically greater intensive care requirements, hospital stays, operative costs, and death with increasing levels of hemodilution, particularly for the lowest value less than 22%. Defoe and colleagues [2] found that patients with a lower hematocrit during cardiopulmonary bypass were associated with a higher risk of in-hospital mortality. However they did not provide data on transfusion or try to separate the effect of transfusion versus anemia on in-hospital mortality. In addition, somewhat unexpectedly, Habib RH [1] also found that hemodilutional anemia on bypass was associated with long-term adverse effects as well. Ongoren MC [6] found that blood transfusion during or after cardiac operation is associated with an increased risk of death over the subsequent five years. He showed that transfused patients also had twice the 5-year mortality (15% vs 7%) of nontransfused patients. After correction for comorbidities and other factors, transfusion was still associated with a 70% increase in mortality. We did not find statistically significant difference for mortality between two groups. All these statistical studies emphasized the importance of blood saving both intra-operative and postoperative period. Many blood precaution techniques has been developed for reducing the risk of intra-operative hemodilution and blood transfusion [8,9]. Predisposing factor for intra-operative hemodilution and transfusion requirement has been described. Multivariate analyses has proved that previous anemia, female gender [13], low BSA, advanced age, CPB time, and renal failure as predictor of intra-operative hemodilution [2-9]. Hemodilution during CPB results from the mixing of pump crystalloid and colloid prime solution with the patient’s blood, and these two relative volumes, along with pre-CPB, will largely determine the nadir value. In addition to above mentioned variables, the type of cardioplegia also effects intra-operative hemodilution. There weren’t any prospective randomized study, identified crystalloid cardioplegia as predictor of hemodilution and transfusion requirement. Eucher PM noticed the hemodilutive effect of crystalloid cardioplegia and described a technique to remove crystalloid cardioplegia by retrograde cardioplegia cannula [14]. We performed this prospective randomized study to identify cristalloid cardioplegia as the predictor of hemodilution. Our multivariate study proved that above mentioned variables (previous anemia, female gender, low BSA, advanced age, CPB time, and renal failure) as predictor of intra-operative hemodilution. But our multivariate analyses also proved that crystalloid cardioplegia is also predictor of intraoperative hemodilution (p < 0.001, OR = 0.19). Transfusion requirement predictors were also well proved. Our study proved that crystalloid cardioplegia is also strong predictor of transfusion requirement (p < 0.001, OR = 0.78). The positive effects of blood cardioplegia are well known. We won’t discuss this preventive ways of blood cardioplegia because of avoiding confounding information.

Another alternative method for applying cardioplegia is minicardioplegia. Its difference from blood cardioplegia consists in the fact that it is applied without diluting the blood in a ratio of 1/4 or 1/8. In the related literature it is possible to find various publications on this topic. In their study, Rıza Türköz et al. found that this method did not necessitate too much volume during pumping and prevented hemodilution from occurring at a high level, while it ensured safe protection of the myocardium [21]. It has been argued in another study that minicardioplegia is more advantageous as regards myocardial edema after ischemic damage [22]. In contrast to these findings, however, there also studies arguing that this method does not provide any advantages over standard blood cardioplegia [23].

## Conclusion

Crystalloid cardioplegia, compared to blood cardioplegia not only causes much more intra-operative hemodilution but also increases the blood transfusion requirement. Hemodilution and increased transfusion increases the ICU and hospital stay, in the early postoperative period.

## Abbreviations

CPB: Cardiopulmonary bypass; BSA: Body surface area; Htc: Hematocrit; RBC: Red blood cell; CABG: Coronary artery bypass graft; OR: Odds ratios; ICU: Intensive care unit.

## Competing interests

The authors declare that they have no competing interests.

## Authors’ contributions

MG, MD = Author. HB, MD = Data collection, Author, Concept/design, Data analysis/interpretation, Critical revision of article, Approval of article. Both authors read and approved the final manuscript.
